# What Kind of Information and Communication Technologies Do Patients with Type 2 Diabetes Mellitus Prefer? An Ecuadorian Cross-Sectional Study

**DOI:** 10.1155/2018/3427389

**Published:** 2018-02-14

**Authors:** Iván Chérrez-Ojeda, Emanuel Vanegas, Erick Calero, Karin Plaza, Jose A. Cano, Juan Carlos Calderon, Jorge Valdano, Jorge Oswaldo Gutierrez, Jose Guevara

**Affiliations:** ^1^Universidad Espíritu Santo, Samborondón, Ecuador; ^2^Respiralab Research Group, Respiralab, Guayaquil, Ecuador

## Abstract

**Purpose:**

The purpose of this study is to assess the frequency of use of information and communication technologies and patterns of preference among Ecuadorian patients with diabetes.

**Methods:**

We conducted an anonymous cross-sectional survey on type 2 diabetes mellitus. A chi-square test for association and adjusted regression analyses were performed.

**Results:**

248 patients were enrolled, with a mean sample age of 57.7 years. SMS was the most used ICT (66.0%). The Internet was used by 45.2% of patients to obtain information about diabetes. SMS and email were rated as the most useful ICTs for receiving information (64.5% and 28.1%, resp.) and asking physicians about diabetes (63.8% and 26.1%, resp.). Patients were also interested in receiving disease information (82.4%) and asking physicians about diabetes (84.7%) through WhatsApp. Adjusted logistic regressions revealed that individuals aged 55 years or younger, those with superior degree level, and those with long diabetes history preferred email for receiving information and asking physicians about diabetes compared to those above 55 years, those with low education level, and those with short diabetes history, respectively.

**Conclusion:**

Understanding preferences of ICTs among patients with diabetes could facilitate application development targeted towards specific requirements from patients.

## 1. Introduction

Diabetes mellitus (DM) is one of the most prevalent chronic diseases worldwide, representing an important cause of mortality and morbidity. In 2014, the NCD Risk Factor Collaboration estimated that 422 million adults were living with diabetes [1]. Latin Americans are an example of the most affected groups by this disease [2]. The prevalence of diabetes in this population is up to 14.3%. Furthermore, 12.4% of adolescents in Ecuador met the criteria for prediabetes [3, 4].

The increase in rates of nonadherence to medication, especially in low socioeconomic populations, has raised concerns about the considerable burden—both societal and individual—of this disease. Therefore, new strategies to improve clinical outcomes involving better communication between healthcare providers and patients are now being explored, giving rise to a heightened interest in the use of information and communication technologies (ICTs) for medical purposes [5–7].

ICTs are broadly defined as technologies used to communicate, manipulate, and store data by electronic means. This concept includes email, SMS text messaging, video chat (i.e., Skype or Hangouts), and online social media (i.e., Facebook or Twitter) as well as all the different computing and mobile-type devices (smartphones and tablets) that perform a wide range of communication and information functions [8, 9]. Recent studies have suggested that the use of ICTs in general may have a positive effect on patients with diabetes by facilitating communication between healthcare professionals and patients, as well as by helping patients learn more about their day-to-day care [10–12].

A recent systematic review and a meta-analysis demonstrated that the use of mHealth significantly improved self-management and glycemic levels measured with HbA1c. Interventions included text messaging, web interfaces, apps, phone calls, computer-based interventions, and social media [10–13]. However, many people stop using a healthcare app shortly after downloading it, suggesting that developers need to address consumer concerns better and that clinical trials are required to test the efficacy of health apps to broaden their appeal and wider use [14–16]. The same concern applies to social media, such as Facebook, where there are profiles that promote therapeutic modalities that are not approved by the FDA and could not be beneficial for a patient with diabetes [17].

Still, little is known about Hispanic population [18]. The aim of this study is to assess the frequency of use of information and communication technologies and patterns of preference among Ecuadorian patients with diabetes. These results might aid in developing new ICTs to promote self-management and improve diabetes outcomes.

## 2. Materials and Methods

We conducted an anonymous cross-sectional survey in which 248 patients with type 2 diabetes from Ecuador rated their typical frequency of ICT usage. Eligible outpatients from either public or private practices in Guayaquil (Ecuador) were surveyed using the Spanish version of the Michigan asthma questionnaire [19]. The survey included 22 items and collected information about demographics, use of cell phones, the interest of patients in using ICTs to receive information about diabetes, and the interest of patients in using ICTs to communicate with healthcare providers about diabetes.

The inclusion criteria employed in this survey were patients older than 18 years who have a diabetes diagnosis of at least 1 year. We excluded patients with psychiatric diseases or language impairment and those who found it difficult to visualize the survey.

### 2.1. Ethical Considerations

The study was reviewed by the ethics committee of Kennedy Hospital in Guayaquil, Ecuador. We obtained informed consent before participation in the survey. We guaranteed that the identities of the patients would not be revealed.

### 2.2. Statistical Analysis

For each ICT, responses to frequency of use were dichotomized into categories of “at least once a week” and “less than once a week.” Age, gender, education level, and years since diabetes diagnosis were used as independent variables on each analysis. Age was categorized into adults (≤55 years of age) and old adults (>55 years of age). Education level was categorized into none/pretertiary and undergraduate/postgraduate. Time since diabetes diagnosis was categorized as ≤8 years and >8 years.

We performed a chi-square test to assess the association among Internet access or owning a cellphone or smartphone and age, gender, education level, and years since diagnosis. We employed the same test to determine the association between same independent variables and the frequency of use of each ICT type and using each ICT to obtain information about their disease.

We performed similar analyses on the associations between the independent variables described and the degree of interest (dichotomized into “high or some interest” and “little or no interest”) in receiving information through each ICT type and interest in communicating (seeking physician) through ICT media type.

We performed adjusted regression analyses between the set of independent variables and use (“at least once a week” and “less than once a week”) and interest in receiving information and communicating through each ICT. Analysis was adjusted for age, gender, education level, and years since diagnosis. Reference categories were >55 years old, male, no education/pretertiary education, and ≤8 years since diagnosis, respectively. Finally, we performed separated nonadjusted analysis between frequency of use of an ICT and interest in receiving information and communicating with physician through that same ICT.

All the data were analyzed using SPSS software, version 24.0 (SPSS Inc., Chicago, IL, USA). We performed Fisher's exact test where necessary. A *p* value of less than 0.05 was considered statistically significant.

## 3. Results

Of the 290 surveys delivered to patients with diabetes, 248 patients filled the survey and were included in this study (nonresponse rate of 14.48%). Mean sample age was 57.7 years (standard deviation [SD]: 10.4). Mean ages of patients ≤55 years and >55 years were 47.75 (SD: 6.02) and 64.38 (SD: 6.65), respectively. Average time with diabetes was 9.4 years (SD: 6.5) and the mean years of diabetes for the ≤8 and >8 years groups were 4.53 (SD: 2.42) and 14.66 (SD: 5.26), respectively. Most patients were female (62.1%) and reported having secondary education (38.7%) (Table 1).

### 3.1. Internet Access and Owning Cellphone or Smartphone

From all, 69.4% reported that they owned a cellphone, of which 46.8% were smartphones and 22.6% were regular mobile phones (Table 2). Internet access was reported by only 27.2% (Table 2). Individuals aged ≤55 were associated with a higher proportion of owning a cellphone and smartphone, as well as having access to Internet (Table 2).

### 3.2. Use of ICTs at Least Once a Week

SMS was the most used ICT (66.0%), followed by the Internet (36.3%), Facebook (31.5%), and email (27.9%). Overall, being 55 years old or younger was associated with a higher proportion of respondents for using every ICT at least once a week compared to patients aged above 55 years (Table 2). Regarding education level, the highest educated patients reported the highest use of ICTs (Supplemental Appendix, Table S2).

### 3.3. Use of ICTs for Seeking Diabetes Information

The Internet was used by 45.2% of patients to obtain information about diabetes. Other ICTs were not used frequently for this purpose (Table 2). Patients with the highest education degree (68.0%) presented a higher proportion of individuals using Internet to seek information than those with no education/pretertiary education (39.0%) (Supplemental Appendix, Table S2). Analysis by years with disease reveals that patients with more than 8 years with diabetes represented the highest frequency of using Internet for this purpose (Supplemental Appendix, Table S3).

### 3.4. High Interest in Receiving Disease Information Using ICTs

**Figure 1:**
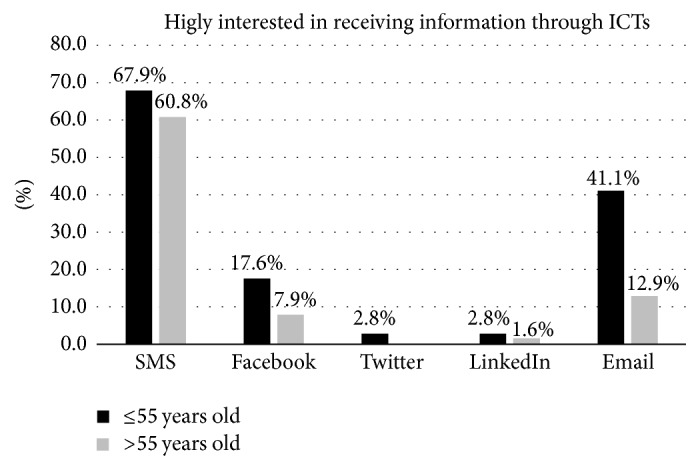
Proportion of patients who are highly interested in receiving information about diabetes through each ICT type.

SMS (64.5%) was the most rated ICT for patients to receive information about their disease, followed by email (28.1%) (Table 2). Regarding gender, males presented the highest proportion of being interested in receiving information through SMS (73.3%) and Facebook (21.4%) (Supplemental Appendix, Table S1). On the other hand, analysis by age and education shows that younger responders with the highest education degree had the highest number of patients interested in receiving information by email (Figure 1) (Table 2; Supplemental Appendix, Table S2).

### 3.5. High Interest in Asking for Physician's Information Using ICT

**Figure 2:**
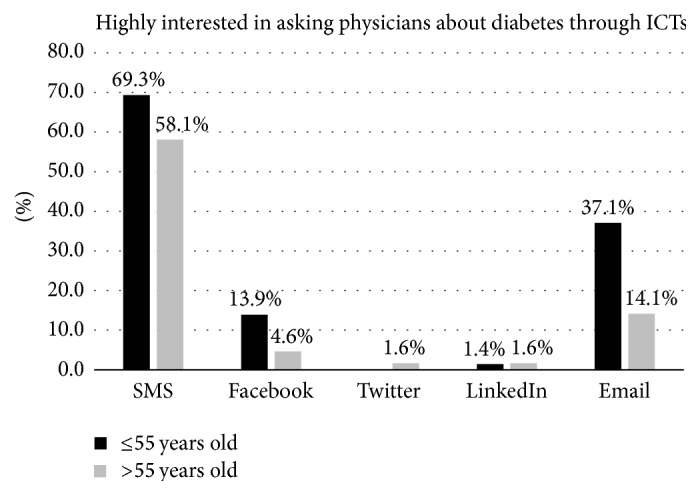
Proportion of patients who are highly interested in asking physicians about diabetes through each ICT type.

SMS and email were rated as useful when it came to asking physicians for information about diabetes (63.8% and 26.1%, resp.) (Table 2). Analysis by age shows that patients with 55 years of age or younger presented the highest rate of being highly interested in communicating with physicians through email (37.1%) (Figure 2 and Table 2). Also males (75.0%) presented a higher percentage of patients with high interest in same subject through SMS than females (54.1%) (Supplemental Appendix, Table S1).

Regarding education level, the highest degree was associated with the greatest proportion of patients being highly interested in requesting information from a physician through SMS and email (81.5 and 45.8%, resp.) (Supplemental Appendix, Table S2).

### 3.6. Interest in Receiving Disease Information and Communicating with Physicians through WhatsApp

A great proportion of patients were interested in receiving disease information (82.4%) and asking physicians about diabetes (84.7%) through WhatsApp. A superior number of patients with 55 years of age or younger (88.6%) were associated with interest in receiving information about diabetes through WhatsApp. Differences between the other variables' categories were not statistically significant.

### 3.7. Logistic Regression Analysis

Adjusted analysis reveals that patients with 55 years of age or younger are more likely to be interested in receiving information about diabetes (OR: 8.77) and communicating with physicians (5.96) through email than those above 55 years of age (reference category) (Table 3). Also responders aged 55 years or younger were more interested in both receiving information (OR: 5.49) and communicating with their physicians (OR: 4.96) using WhatsApp than patients above 55 years of age (Table 3).

Furthermore, females were associated with a lesser chance of being highly interested in receiving information through Facebook (OR: 0.21) and communicating with physicians through SMS (OR: 0.42) than males (reference category) (Table 3). Moreover, patients with superior degree presented greater odds of being interested in receiving information about their disease through email (OR: 4.32) than those with no schooling or pretertiary education (reference category) (Table 3).

In addition, a greater likelihood of being highly interested in receiving information about disease (OR: 2.70) and communicating with physicians (OR: 2.92) through email was associated with patients diagnosed with diabetes for more than 8 years when compared to those having the disease for 8 years or less (reference category) (Table 3). However, these patients were also less likely to be highly interested in receiving information through Facebook (OR: 0.25) than the reference.

Finally, nonadjusted regressions found that responders that used SMS, Facebook, and email at least once a week were more likely to be highly interested in receiving disease information and communicating with physicians through these same ICTs when compared to responders using the ICT less than once a week (reference category) (Table 3).

If an individual indicated no interest in using an ICT to receive information or communicate with their physician, we included open-ended questions to explain why. However, only 0.40%  (*n* = 1) was not interested in receiving information through any type of ICT, while all patients were interested in at least one ICT for asking physicians about diabetes. The patient that was not interested in receiving information through any ICT type explained that she preferred to communicate directly with her physician through phone calls or personally.

## 4. Discussion

Essential DM self-management is generally divided into different domains: nutritional management, exercise and physical activity, blood glucose monitoring and medication utilization, risk reduction, problem solving, and healthy coping [20, 21].

However, DM patients who do not receive education may suffer from many complications because of poor self-management [22, 23]. This is where information and communication technologies may play a major role to improve the clinical condition of patients. The rapid diffusion, low cost, and general availability of ICTs make them an attractive platform for managing care, communication, and interventions in chronic diseases as part of Self-Management Education (SME). For instance, telehealth interventions have shown positive effects on improving the DM control self-management in primary healthcare settings [24]. Anyway, in the present study, we found that 72.8% of patients have no access to Internet, and less than half have a smartphone. These results highlight the fact that access to technology in Latin America is restricted to a small group, which represents a limitation for health interventions through ICTs.

It has been reported that Latinos living in California make greater use of SMS, email, and Facebook than any other ICT [25, 26]. The present study suggests that SMS and email were the most useful tools for all patients for receiving DBT information and communicating with physicians, regardless of age, gender, education, or years with disease. There are several practical reasons for using SMS. It costs less than voice messaging, and it can reach people whose phones are switched off. Furthermore, SMS messaging is silent, which means that it can be used in places where it may be impractical to hold a conversation [27].

Regarding email, we found that patients with a diagnosis of DM of more than 8 years were approximately 3 times more likely to be interested in receiving information and communicating with physicians through email. Our findings are similar to those reported by Giménez-Pérez et al., where patients with a type I DM diagnosis of 18.4 years prefer communicating with healthcare professionals through email instead of Facebook [28]. In both studies, the population subject to analysis was above the second half of adulthood. Even though we found that patients above 55 years of age were less likely to use ICTs for these purposes, the preference of using only email in this group could be due to aging and chronicity of the disease, which predispose patients to become isolated from society, and feeling of loneliness [29]. Likewise, in Giménez-Pérez et al.'s study, patients in the fourth quartile of age (52 y–75 y) presented the lowest proportion of willingness to share information online with professionals. These data support the fact that a long history of chronic disease in the setting of advanced age favors the development of loneliness. A systematic review of loneliness and common chronic physical conditions in adults establishes how such behavior can be a significant biopsychosocial stressor that can complicate the course of the disease through reciprocal relationship with outcomes of diabetes [30].

WhatsApp is a cross-platform instant messaging application that allows smartphone users to exchange text, image, video, and audio messages for free. In Latin America, around two-thirds of Internet users are now “WhatsApping” in contrast with North Americans who have a somewhat lesser frequency of use of WhatsApp [31]. Our results suggest that WhatsApp could be appropriate for patients aged 55 or younger since they are five times more likely to receive information and communicate with physician through this ICT than others. Owing to the different categories of interest used in analysis for WhatsApp and other electronic networks, they cannot be directly compared.

Currently, in Latin America, some mobile operators offer different plans that include unlimited data usage exclusively for WhatsApp and Facebook services. This is probably one of the reasons why Latin America has a high social media penetration rate compared with other global regions [31]. We found among participants that Facebook was used as the third option for receiving and communicating with physicians, though it was a small proportion of patients.

There are different Facebook support groups, where patients can share their stories, learn from others, increase their knowledge, and build up hope. In a systematic review, examining the benefits of social media in chronic diseases, 45% of studies identified a positive impact with respect to support (via blogs) and disease modification (via Facebook) [9]. Furthermore, two qualitative studies about diabetes-related Facebook groups conducted by Greene et al. reported that more than 60% of wall-post topics were based on sharing information about diabetes [17, 32].

Level of education and income might influence ICT use in Hispanics. Hispanics with more education and with higher household incomes are more likely to report using the Internet. As a matter of fact, in 2015, only two-thirds of Hispanics (67%) with an education degree lower than high school reported being online, while 95% of those with at least some college experience were online during the year [33]. Our results show a similar pattern, where patients with higher education levels presented a higher proportion of using every ICT more frequently, looking for information through such ICTs, and being interested in receiving and communicating with physicians than those with the lowest education degree.

Our results have important implications for designing interventions using ICTs to improve patient education and communication between healthcare professionals and patients. Given the fact that most of the population in Latin America belongs to a low socioeconomic status and public healthcare is not equally accessible to all individuals, it is imperative to find methods to diffuse medical advice efficiently. In developing nations, people living in urban areas have a reasonable chance of access to medical care, while those in semiurban or rural areas do not have such chance. If available, the former group may have access to very limited, if not deficient, healthcare. Our results suggest that email and SMS were used by patients in every age group. Also other social media channels, such as WhatsApp and Facebook, are becoming increasingly popular among patients. Even though it has been reported that there is a significant difference in preferences of health information technology tools used between rural and urban citizens in the United States, there is no current information on whether such observation replicates in Latin America [34].

Our study has some limitations. First, it was not conducted in all Latin American countries, and the preferred use of ICTs in other countries could differ. Our participants knew the purpose of the study, which may have affected the answers some of them gave. Our survey has not been validated, and our results could lead to biased or inaccurate conclusions. Finally, due to missing data in the surveys that were left blank by patients who did not want to fill the study survey, our group could not establish whether any significant differences existed between the respondent and nonrespondent groups.

However, one strength point of this study is that it covered a reasonably large sample size of DM patients. The sample included participants of different age, gender, and education level and, to the best of our knowledge, our study is the first to explore the use and preference of ICTs in Latin American DM patients.

Future research is needed to confirm our findings and assess the real use of ICTs tools as an appropriate development of mobile health applications. Any programmatic solution must be accompanied by a distribution strategy to increase patient acceptance and use of health care-related applications. Physicians should be enrolled with app developers to evaluate the design and development of such tools.

## 5. Conclusion

There are different preferences of ICTs in type 2 DM patients. SMS and email were the most useful tools for all patients for receiving DBT information and communicating with physicians. A higher education degree is associated with a higher interest of using ICTs for health-related purposes. The widespread use of ICTs opens new possibilities to the relationship between physicians and patients, improving channels of communication that go in both directions.

## Figures and Tables

**Table 1:** Demographic information of surveyed population.

Characteristics	Patients (*n* = 248) *n* (%)
*Age (years)*	
≤55 years old	100 (40.3)
>55 years old	148 (59.7)
*Gender*	
Male	89 (35.9)
Female	154 (62.1)
*Race*	
White	14 (5.6)
Native	0 (0)
Hispanic/mestizo	220 (88.7)
Black	7 (2.8)
Other	0 (0)
*Education level*	
None	30 (12.1)
Primary school	86 (34.7)
Secondary school	96 (38.7)
Undergraduate	32 (12.9)
Postgraduate	1 (0.4)
*Years with diabetes*	
≤8	127 (51.2)
>8	116 (46.8)

**Table 2:** Use, obtaining information, interest in receiving information, and interest in asking a physician through ICT types by age.

	≤55 years old (*n* = 100)	>55 years old (*n* = 148)	Chi-square *p* value	Total (*n* = 248)
Internet access	48.0	13.0	.000	27.2
*Owning*				
Cellphone	90.0	55.4	.000	69.4
Smartphone	64.3	27.0	.000	46.8
*Use of ICT type (at least once a week)*				
SMS	75.6	55.8	.009	66.0
Facebook	45.9	15.9	.000	31.5
Twitter	20.8	4.4	.004	12.9
YouTube	38.4	8.8	.000	24.1
Email	41.7	13.2	.000	27.9
Internet	52.0	19.7	.000	36.3
LinkedIn	5.6	0.0	.120^a^	2.9
Skype	6.9	1.5	.211^a^	4.3
*Uses ICT to obtain information about disease*				
Internet	54.7	35.2	.018	45.2
Facebook	8.3	2.9	.275^a^	5.6
Twitter	4.2	1.4	.620^a^	2.8
YouTube	4.2	1.4	.620^a^	2.8
Email	15.3	4.3	.028	9.9
*Interest in receiving information through ICT type (high/some interest)*				
SMS	67.9	60.8	.358	64.5
Facebook	17.6	7.9	.096	13.1
Twitter	2.8	0.0	.499^a^	1.5
LinkedIn	2.8	1.6	1.000^a^	2.2
Email	41.1	12.9	.000	28.1
*Interest in asking physician through ICT type (high/some interest)*				
SMS	69.3	58.1	.154	63.8
Facebook	13.9	4.6	.064	9.5
Twitter	0.0	1.6	.478^a^	0.7
LinkedIn	1.4	1.6	1.000^a^	1.5
Email	37.1	14.1	.002	26.1
*Interest in receiving information through WhatsApp (yes/no)*				
Interested	88.9	65.0	.034^a^	82.4
*Interest in asking physician about disease through WhatsApp (yes/no)*				
Interested	90.6	68.4	.056^a^	84.7

*Notes*. All data are presented as percentages. Differences in values between the two age groups are significant at .05 significance level. ^a^Fisher's exact test performed.

**Table 3:** Characteristics of frequent users of selected ICT types (≥1 X/week) showing high/some interest in receiving information and asking physicians about diabetes.

Variable	Interest in receiving information through ICT type OR (95% CI)	Interest in asking physicians through ICT type OR (95% CI)
*SMS *(*n* = 159)		
Gender^a^		
Female	0.49 (0.24–1.01)	**0.42 (0.20**–**0.89)**
Weekly SMS use^b^	**7.52 (3.49**–**16.24)**	**7.33 (3.37**–**15.94)**
*Facebook *(*n* = 143)		
Gender		
Female	**0.21 (0.07**–**0.69)**	0.77 (0.23–2.55)
Years with diabetes^c^		
>8 years	**0.25 (0.07**–**0.90)**	0.33 (0.08–1.30)
Weekly Facebook use	**14.50 (3.92**–**53.59)**	**8.89 (2.30**–**34.32)**
*Email *(*n* = 140)		
Age^d^		
≤55 years old	**8.77 (2.73**–**28.20)**	**5.96 (1.96**–**18.16)**
Education level^e^		
Undergraduate/postgraduate	**4.32 (1.39**–**13.39)**	2.75 (0.91–8.31)
Years with diabetes		
>8 years	**2.70 (1.02**–**7.17)**	**2.92 (1.07**–**7.94)**
Weekly email use	**47.05 (15.75**–**140.59)**	**25.47 (9.29**–**69.85)**
*WhatsApp*		
Age^a^		
≤55 years old	**5.49 (1.42**–**21.20)**	**4.96 (1.16**–**21.30)**

*Notes*. Regression analysis was adjusted for variables such as age, gender, education level, and years with diabetes. Regression analysis using weekly ICT type was performed separately (nonadjusted). Bolded values are significant at 0.05 significance level. OR, odds ratio; CI, confidence interval. ^a^Reference “gender” category is male. ^b^Reference “ICT use” category is “less than once a week.” ^c^Reference “years with diabetes” category is ≤8 years. ^d^Reference “age” category is >55 years old. ^e^Reference “educational level” category is no education/pretertiary.

## Supplementary Materials

Supplementary Materials Table S1: use, obtaining information, interest in receiving information, and interest in asking a physician through ICT types by gender. Table S2: use, obtaining information, interest in receiving information, and interest in asking a physician through ICT types by education level. Table S3: use, obtaining information, interest in receiving information, and interest in asking a physician through ICT types by years since diagnosis.
