# Supplementary material for: What Kind of Information and Communication Technologies Do Patients with Type 2 Diabetes Mellitus Prefer? An Ecuadorian Cross-Sectional Study
Source: Int J Telemed Appl. 2018 Feb 14;2018:3427389. doi: 10.1155/2018/3427389 (PMC5832117; doi:10.1155/2018/3427389)
Supplement: Supplementary Materials — Table S1: use, obtaining information, interest in receiving information, and interest in asking a physician through ICT types by gender. Table S2: use, obtaining information, interest in receiving information, and interest in asking a physician through ICT types by education level. Table S3: use, obtaining information, interest in receiving information, and interest in asking a physician through ICT types by years since diagnosis. [file 3427389.f1.docx]

**Supplemental Appendix**

| Table S1 – Use, obtain information, interest in receiving information, and interest in asking a physician through ICT types by gender. | | | | |
| --- | --- | --- | --- | --- |
|  | Male  (n=89) | Female  (n=154) | Chi-square p-value | Total  (n=243) |
| Internet Access | 36.0% | 21.1% | .011 | 26.6% |
| **Owning** | | | | |
| Cellphone | 75.3% | 65.6% | .115 | 69.1% |
| Smartphone | 53.1% | 41.1% | .140 | 46.1% |
| **Use of ICT type (at least once a week)** | | | | |
| SMS | 78.7% | 56.4% | .004 | 65.2% |
| Facebook | 37.9% | 25.9% | .131 | 30.9% |
| Twitter | 12.5% | 11.3% | .824 | 11.8% |
| YouTube | 29.8% | 18.8% | .131 | 23.4% |
| Email | 30.4% | 23.8% | .390 | 26.5% |
| Internet | 47.5% | 26.5% | .010 | 35.2% |
| LinkedIn | 1.8% | 2.6% | 1.000^a^ | 2.2% |
| Skype | 3.6% | 5.1% | 1.000^a^ | 4.5% |
| **Uses ICT to obtain information about disease** | | | | |
| Internet | 50.0% | 39.5% | .210 | 44.1% |
| Facebook | 0.0% | 8.9% | .020^a^ | 5.1% |
| Twitter | 1.7% | 3.8% | .636^a^ | 2.9% |
| YouTube | 1.7% | 3.8% | .636^a^ | 2.9% |
| Email | 6.8% | 10.1% | .490 | 8.7% |
| **Interest in receiving information through ICT type (high/some interest)** | | | | |
| SMS | 73.3% | 56.8% | .040 | 63.5% |
| Facebook | 21.4% | 7.8% | .023 | 13.5% |
| Twitter | 1.9% | 1.3% | 1.000^a^ | 1.5% |
| LinkedIn | 3.7% | 1.3% | .570^a^ | 2.3% |
| Email | 30.9% | 25.0% | .455 | 27.5% |
| **Interest in seeking physician through ICT type (high/some interest)** | | | | |
| SMS | 75.0% | 54.1% | .010 | 62.8% |
| Facebook | 10.7% | 9.1% | .756 | 9.8% |
| Twitter | 0.0% | 1.3% | 1.000^a^ | 0.8% |
| LinkedIn | 0.0% | 1.3% | 1.000^a^ | 0.8% |
| Email | 29.1% | 21.3% | .310 | 24.6% |
| **Interest in receiving information through WhatsApp (Yes/No)** | | | | |
| Interested | 82.9% | 80.6% | .802 | 81.7% |
| **Interest in seeking physician about disease through WhatsApp (Yes/No)** | | | | |
| Interested | 87.9% | 80.6% | .406 | 84.1% |
| Notes: All data are presented as percentages. Differences in values between the two gender groups are significant at .05 significance level. ^a.^ Fisher exact test performed. | | | | |

| Table S2 – Use, obtain information, interest in receiving information, and interest in asking a physician through ICT types by education level. | | | | |
| --- | --- | --- | --- | --- |
|  | No education/  Pre-tertiary  (n=212) | Undergraduate/  Postgraduate  (n=33) | Chi-square p-value | Total  (n=245) |
| Internet Access | 22.4% | 51.5% | .000 | 26.3% |
| **Owning** | | | | |
| Cellphone | 66.5% | 84.8% | .034 | 69.0% |
| Smartphone | 41.7% | 64.3% | .030 | 45.8% |
| **Use of ICT type (at least once a week)** | | | | |
| SMS | 60.3% | 92.0% | .002 | 65.4% |
| Facebook | 24.1% | 58.3% | .001 | 30.0% |
| Twitter | 8.8% | 20.8% | .141^a^ | 10.9% |
| YouTube | 17.5% | 45.8% | .003 | 22.5% |
| Email | 20.4% | 54.2% | .001 | 26.3% |
| Internet | 29.9% | 57.7% | .007 | 35.0% |
| LinkedIn | 1.8% | 4.3% | .429^a^ | 2.2% |
| Skype | 2.7% | 4.3% | .531^a^ | 3.0% |
| **Uses ICT to obtain information about disease** | | | | |
| Internet | 39.0% | 68.0% | .008 | 44.1% |
| Facebook | 3.5% | 12.5% | .099^a^ | 5.0% |
| Twitter | 1.7% | 4.2% | .436^a^ | 2.2% |
| YouTube | 1.7% | 4.2% | .436^a^ | 2.2% |
| Email | 7.8% | 20.8% | .068^a^ | 10.1% |
| **Interest in receiving information through ICT type (high/some interest)** | | | | |
| SMS | 61.5% | 77.8% | .109 | 64.4% |
| Facebook | 11.7% | 17.4% | .493^a^ | 12.7% |
| Twitter | 0.9% | 0.0% | 1.000^a^ | 0.8% |
| LinkedIn | 2.8% | 0.0% | 1.000^a^ | 2.3% |
| Email | 21.1% | 52.2% | .002 | 26.5% |
| **Interest in seeking physician through ICT type (high/some interest)** | | | | |
| SMS | 60.5% | 81.5% | .040 | 64.4% |
| Facebook | 9.1% | 12.5% | .702^a^ | 9.7% |
| Twitter | 0.0% | 0.0% | N/A^b^ | 0.0% |
| LinkedIn | 0.0% | 4.2% | .185^a^ | 0.8% |
| Email | 19.6% | 45.8% | .007 | 24.4% |
| **Interest in receiving information through WhatsApp (Yes/No)** | | | | |
| Interested | 82.7% | 78.9% | .736^a^ | 81.7% |
| **Interest in seeking physician about disease through WhatsApp (Yes/No)** | | | | |
| Interested | 82.7% | 88.2% | .719^a^ | 84.1% |
| Notes: All data are presented as percentages. Differences in values between the two education level groups are significant at .05 significance level. ^a.^ Fisher exact test performed.  ^b.^ Variable is a constant, no test performed. | | | | |

| Table S3 – Use, obtain information, interest in receiving information, and interest in asking a physician through ICT types by years since diagnosis | | | | |
| --- | --- | --- | --- | --- |
|  | ≤ 8 years with diabetes  (n=127) | > 8 years with diabetes  (n=116) | Chi-square p-value | Total  (n=243) |
| Internet Access | 28.0% | 25.9% | .709 | 27.0% |
| **Owning** | | | | |
| Cellphone | 74.0% | 63.8% | .085 | 69.1% |
| Smartphone | 50.6% | 40.8% | .226 | 46.1% |
| **Use of ICT type (at least once a week)** | | | | |
| SMS | 67.1% | 62.9% | .585 | 65.2% |
| Facebook | 38.7% | 23.4% | .054 | 31.7% |
| Twitter | 11.1% | 14.1% | .603 | 12.5% |
| YouTube | 26.0% | 21.9% | .571 | 24.1% |
| Email | 27.8% | 26.6% | .874 | 27.2% |
| Internet | 36.0% | 35.8% | .982 | 35.9% |
| LinkedIn | 4.1% | 1.6% | .624^a^ | 3.0% |
| Skype | 5.5% | 3.3% | .688^a^ | 4.5% |
| **Uses ICT to obtain information about disease** | | | | |
| Internet | 32.9% | 55.1% | .008 | 43.7% |
| Facebook | 8.2% | 3.1% | .281^a^ | 5.8% |
| Twitter | 1.4% | 3.1% | .601^a^ | 2.2% |
| YouTube | 4.1% | 1.5% | .622^a^ | 2.9% |
| Email | 13.7% | 4.6% | .068 | 9.4% |
| **Interest in receiving information through ICT type (high/some interest)** | | | | |
| SMS | 58.4% | 69.0% | .182 | 63.5% |
| Facebook | 18.3% | 8.1% | .085 | 13.5% |
| Twitter | 1.4% | 1.6% | 1.000^a^ | 1.5% |
| LinkedIn | 2.9% | 1.6% | 1.000^a^ | 2.3% |
| Email | 21.7% | 32.3% | .174 | 26.7% |
| **Interest in seeking physician through ICT type (high/some interest)** | | | | |
| SMS | 57.9% | 68.1% | .204 | 62.8% |
| Facebook | 14.3% | 4.8% | .065 | 9.8% |
| Twitter | 0.0% | 1.6% | .477^a^ | 0.8% |
| LinkedIn | 1.5% | 1.6% | 1.000^a^ | 1.6% |
| Email | 19.4% | 30.2% | .155 | 24.6% |
| **Interest in receiving information through WhatsApp (Yes/No)** | | | | |
| Interested | 85.0% | 77.4% | .413 | 81.7% |
| **Interest in seeking physician about disease through WhatsApp (Yes/No)** | | | | |
| Interested | 87.2% | 80.0% | .514^a^ | 87.2% |
| Notes: All data are presented as percentages. Differences in values between the two “years since diagnosis” groups are significant at .05 significance level. ^a.^ Fisher exact test performed. | | | | |
